# The deficient CLEC5A ameliorates the behavioral and pathological deficits via the microglial Aβ clearance in Alzheimer’s disease mouse model

**DOI:** 10.1186/s12974-024-03253-x

**Published:** 2024-10-23

**Authors:** Yu-Yi Lin, Wen-Han Chang, Shie-Liang Hsieh, Irene Han-Juo Cheng

**Affiliations:** 1 Institute of Brain Science, National Yang Ming Chiao Tung University, Taipei, Taiwan; 2 Brain Research Center, National Yang Ming Chiao Tung University, Taipei, Taiwan; 3 Immunology Research Center, National Health Research Institutes, Zhunan, Taiwan; 4 Institute of Clinical Medicine, Institute of Microbiology and Immunology, National Yang Ming Chiao Tung University, Taipei, Taiwan; 5 Department of Medical Research, Taipei Veterans General Hospital, Taipei, Taiwan

**Keywords:** Alzheimer’s disease, β-amyloid (Aβ), Microglia, C-type lectin domain family 5 member A (CLEC5A)

## Abstract

**Background:**

Alzheimer’s disease (AD) is a neurodegenerative disease that causes cognitive dysfunction in older adults. One of the AD pathological factors, β-Amyloid (Aβ), triggers inflammatory responses and phagocytosis of microglia. C-type lectin domain family 5 member A (CLEC5A) induces over-reactive inflammatory responses in several virus infections. Yet, the role of CLEC5A in AD progression remains unknown. This study aimed to elucidate the contribution of CLEC5A to Aβ-induced microglial activation and behavioral deficits.

**Methods:**

The AD mouse model was crossed with *Clec5a* knockout mice for subsequent behavioral and pathological tests. The memory deficit was revealed by the Morris water maze, while the nociception abnormalities were examined by the von Frey filament and hotplate test. The Aβ deposition and microglia recruitment were identified by ELISA and immunohistochemistry. The inflammatory signals were identified by ELISA and western blotting. In the *Clec5a* knockdown microglial cell model and *Clec5a* knockout primary microglia, the microglial phagocytosis was revealed using the fluorescent-labeled Aβ.

**Results:**

The AD mice with *Clec5a* knockout improved Aβ-induced memory deficit and abnormal nociception. These mice have reduced Aβ deposition and increased microglia coverage surrounding the amyloid plaque, suggesting the involvement of CLEC5A in AD progression and Aβ clearance. Moreover, the phagocytosis was also increased in the Aβ-stressed *Clec5a* knockdown microglial cell lines and *Clec5a* knockout primary microglia.

**Conclusion:**

The *Clec5a* knockout ameliorates AD-like deficits by modulating microglial Aβ clearance. This study implies that targeting microglial *Clec5a* could offer a promising approach to mitigate AD progression.

**Supplementary Information:**

The online version contains supplementary material available at 10.1186/s12974-024-03253-x.

## Introduction

Alzheimer’s disease (AD) is the primary cause of cognitive decline, primarily affecting individuals over 65. Despite recent advances in treatments [1], the underlying causes of the disease remain elusive. The β-amyloid (Aβ) peptide, a proteolytic cleavage product of amyloid precursor protein (APP), is one of the pathological hallmarks of AD [2]. The Aβ peptide typically consists of 40 or 42 amino acids, referred to as Aβ_1−40_ and Aβ_1−42_, respectively. The longer variant Aβ_1−42_ is more susceptible to aggregate [3, 4]. The Aβ peptides can aggregate into dimers, trimers, oligomers, protofibrils, and amyloid fibrils [5]. Abnormal aggregation and accumulation of Aβ in the brain triggers neurodegeneration and neuroinflammation, leading to cognitive impairments [6].

Aggregated Aβ activates microglia into diverse phenotypes, exerting detrimental or protective roles in different disease stages [7–9]. The detrimental role of microglia through the pro-inflammatory response and aberrant neuronal pruning results in neurodegeneration and memory decline in AD [10]. The pro-inflammatory cytokines are increased in the brains of AD patients and Aβ-stimulus human microglial cell lines [11–14]. The Aβ activates NOD-like receptor protein 3 (NLRP3) inflammasome to trigger the production of pro-inflammatory molecules [15, 16]. The depletion of NLRP3 attenuates the memory decline and Aβ aggregates in the AD mouse model [17]. On the other hand, reactive microglia have higher phagocytic activity to clear abnormal proteins and facilitate tissue repair in several brain diseases [18, 19]. In the AD mouse model, enhanced microglial phagocytosis reduces the Aβ burden and alleviates memory declines [20–23], while suppressed microglial phagocytosis aggravates the Aβ pathology [20, 24]. Therefore, modulating microglia’s inflammatory phenotypes and Aβ clearance ability are potential avenues for slowing AD progression.

C-type lectin domain family 5 member A (CLEC5A) is a spleen tyrosine kinase (Syk)-coupled pattern recognition receptor in myeloid cells [25]. The Syk signaling regulates NLRP3 to modulate Aβ-induced inflammatory responses and Aβ clearance in microglia [26–28]. CLEC5A mediates inflammatory signals after the infection of dengue virus, Japanese encephalitis virus (JEV), influenza virus, and severe acute respiratory syndrome coronavirus 2 [29–32]. The *Clec5a* level was dramatically elevated in the mouse brain after JEV infection [33], and the ratio of CLEC5A^+^ cells was increased in the liver of the hepatitis mouse model [34]. After virus infection, administration of CLEC5A-blocking antibodies inhibits the NLRP3 inflammasome activation [35] and reduces the release of pro-inflammatory cytokines [30]. Similarly, *Clec5a* knockdown by shRNA displays reduces pro-inflammatory responses [29]. However, the participation of CLEC5A in Aβ-induced microglial responses remains enigmatic.

This research investigates the role of CLEC5A on Aβ-induced deficits. In AD mice with *Clec5a* knockout, spatial memory deficits and Aβ accumulation were reversed. *Clec5a* knockdown microglia exhibited increased phagocytosis and the degreased NLRP3 inflammasome. Therefore, inhibiting CLEC5A may be a potential approach to alleviate the Aβ-induced cognitive impairments.

## Results

**Fig. 1:**
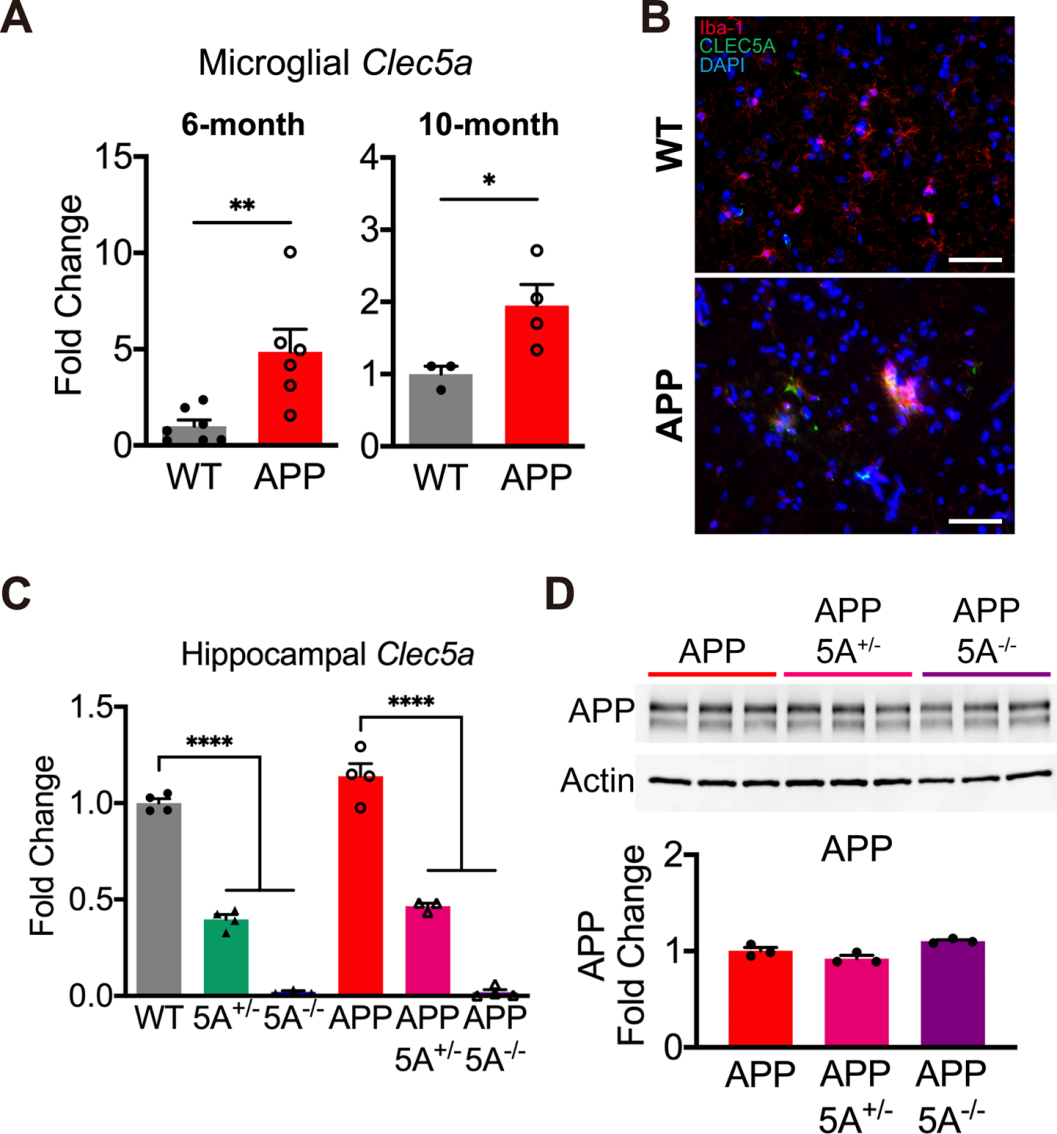
Knockout the *Clec5a* gene in the AD mouse model. (**A**) The levels of microglial *Clec5a* mRNA in 6-month-old (left panel) and 10-month-old (right panel) WT and APP mice were analyzed by qPCR and normalized to the level of *Iba-1* mRNA. *n* = 6–7 mice in 6-month-old mice. *n* = 3–4 mice in 10-month-old mice. (**B**) The double staining of CLEC5A *(green)* and Iba-1 *(red)* in WT and APP mouse hippocampus. Scale bar: 50 μm. (**C**) The *Clec5a* mRNA levels in WT, 5A^+/−^, 5A^−/−^, APP, APP5A^+/−^ and APP5A^−/−^ were measured by qPCR. *n* = 4 for WT, 5A^+/−^, APP, and APP5A^−/−^, *n* = 3 for 5A^−/−^ and APP5A^+/−^. (**D**) The Western blot image (upper panel) and quantification (lower panel) of hippocampal APP protein levels in APP, APP5A^+/−^ and APP5A^−/−^ mice. *n* = 3 for each group. *p ≤ 0.05, **p ≤ 0.01, ****p ≤ 0.0001

*Clec5a* is mainly expressed in the microglia in the brain according to the Human Protein Atlas, Protein Atlas version 23.0 (proteinatlas.org) [36] (Fig. S1A). To investigate the *Clec5a* alterations in AD, we isolated hippocampal microglia from 6- and 10-month-old *APP* transgenic mice and their wild-type (WT) littermates. The qPCR analysis indicated that *Clec5a* mRNA levels were higher in *APP* transgenic mice than WT mice (Fig. 1A), resembling the response observed following viral infection [33, 34]. Immunohistochemistry analysis also indicated that CLEC5A protein is more readily detectable in the microglia of 6-month-old *APP* transgenic mice than in WT control mice (Fig. 1B). We speculated that the increase of *Clec5a* mediates neuroinflammation and thus accelerates AD pathogenesis in APP mice.

To investigate the role of *Clec5a* in AD, *Clec5a* homozygous knockout (5A^−/−^) mice were crossed with *APP* hemizygous transgenic mice to generate *Clec5a* heterozygous knockout mice with (APP5A^+/−^) or without (5A^+/−^) *APP* transgene. Next, APP5A^+/−^ mice were intercrossed with 5A^+/−^ mice to generate six genotypes of offspring: WT, 5A^+/−^, 5A^−/−^, APP, and APP5A^+/−^ and APP5A^−/−^ (Fig. S1B). The qPCR analysis demonstrated a reduction in *Clec5a* mRNA expression corresponding to decreased gene copy numbers (Fig. 1C). The Western blot analysis indicated that the APP protein level did not alter in *APP* transgenic mice with the *Clec5a* knockout (Fig. 1D). For all the subsequent analyses, four genotypes of littermate mice were utilized: WT, 5A^−/−^, APP, and APP5A^−/−^.

### *Clec5a* knockout reverses memory and nociception deficits

**Fig. 2:**
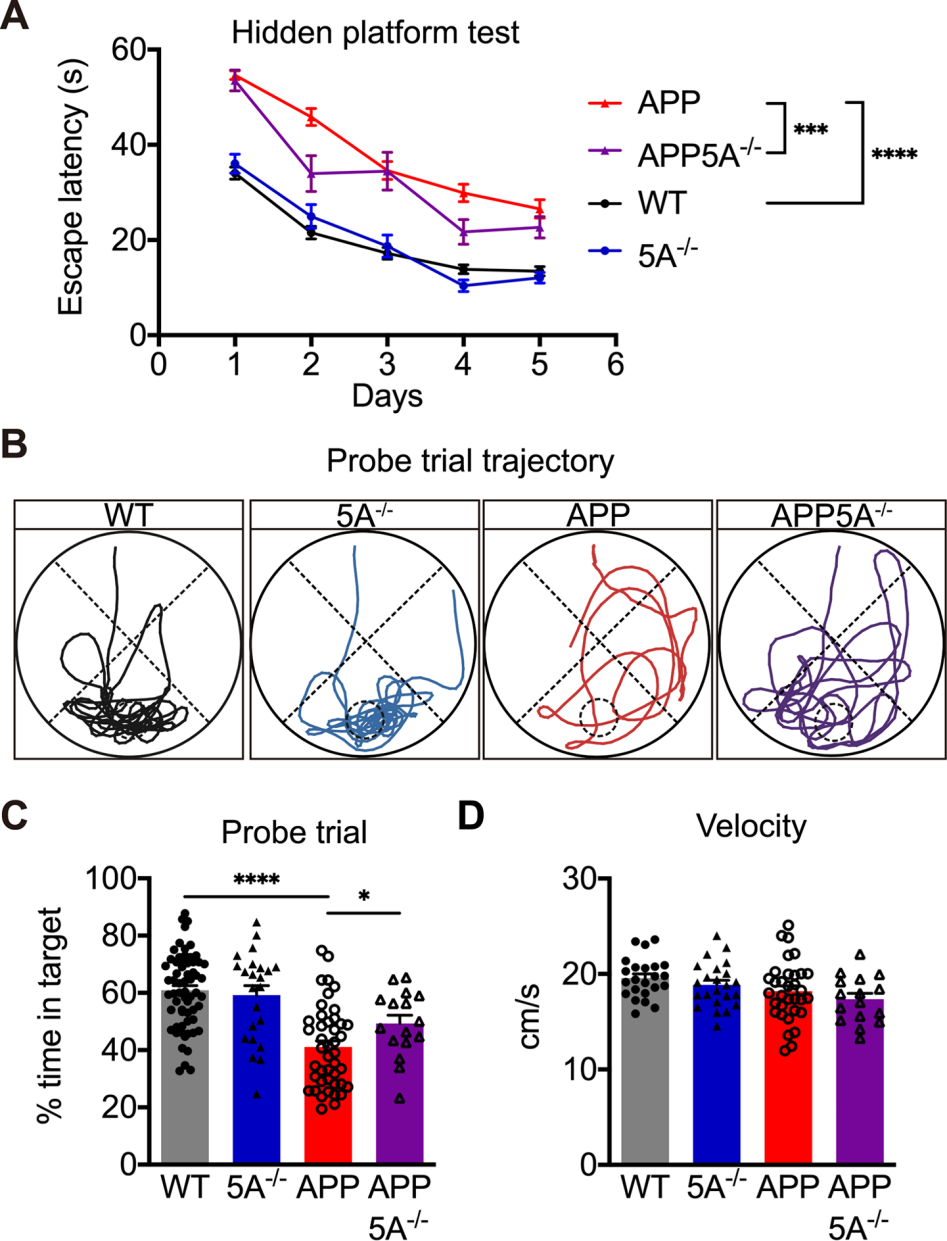
*Clec5a* knockout improves the learning and memory deficit of the AD mouse model in the Morris water maze. (**A**) The mean daily escape latency in the 5-day hidden platform trial. (**B**) The representative paths are depicted during the probe trial. The dashed circle indicates the previous position of the platform. The pool is divided into four quadrants, delineated by dashed lines. The area where the platform was initially located is defined as the target quadrant. (**C**) The percentage of time spent in the target quadrant in the probe trial. (**D**) The swimming speed during the probe trial. *n* = 59 for WT, *n* = 42 for 5A^−/−^, *n* = 23 for APP, *n* = 16 for APP5A^−/−^. *p ≤ 0.05, ***p ≤ 0.001, ****p ≤ 0.0001

The *APP* transgenic mouse model used in this study develops spatial memory impairment after 4 months of age [37]. The Morris water maze test was performed to examine spatial learning and memory in 6-month-old mice. In a 5-day hidden platform training, the APP mice had significantly longer escape latency than WT mice, indicating a deficiency in memory acquisition. The APP5A^−/−^ mice exhibited shorter escape latency than APP mice, suggesting that the *Clec5a* knockout improved the memory acquisition deficit in APP mice. The 5A^−/−^ mice showed no significant alteration in escape latency compared to WT mice (Fig. 2A).

The probe trial assessed the memory retention of the mice by monitoring their swimming behavior in the pool without the platform for 60 s. The APP mice spent significantly less time in the target quadrant than WT mice, indicating a deficit of memory retention in APP mice. The APP5A^−/−^ mice spent a significantly longer time in the target quadrant than APP mice, but 5A^−/−^ mice performed as well as WT mice (Fig. 2B, C). These results suggested that *Clec5a* knockout in the AD mouse model improves their memory retention deficit at six months of age. There was no difference in swimming speed between the groups, suggesting that *Clec5a* knockout does not affect motor function (Fig. 2D).

**Fig. 3:**
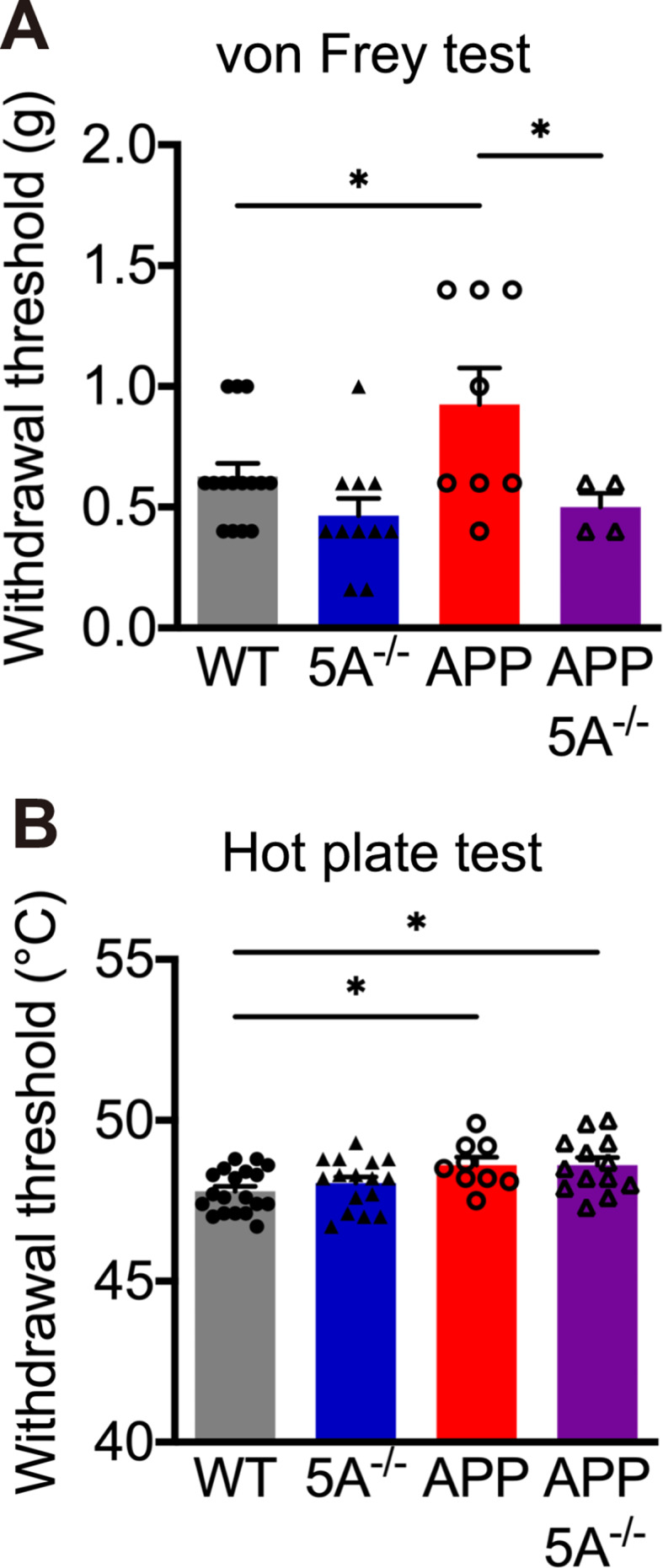
*Clec5a* knockout ameliorates the mechanical but not thermal nociception deficits in the AD mouse model. (**A**) The von Frey filament test measured the threshold of mechanical nociception. *n* = 15 for WT, *n* = 11 for 5A^−/−^, *n* = 8 for APP, *n* = 4 for APP5A^−/−^. (**B**) The hot plate test measured the threshold of thermal sensation. *n* = 19 for WT, *n* = 16 for 5A^−/−^, *n* = 9 for APP, *n* = 13 for APP5A^−/−^. *p ≤ 0.05

We previously reported that *APP* transgenic mice are less sensitive to nociceptive stimulation [38]. In addition, aberrant microglial activation has been associated with changes in nociception sensitivity [39–41]. To investigate the role of *Clec5a* on nociception, we examined the thermal and mechanical sensations of these 4 groups of mice by von Frey filaments and hot plate tests. In the von Frey test, APP mice required higher force to induce paw withdrawal than WT mice (Fig. 3A), which suggested that APP mice had higher mechanical sensation thresholds. Meanwhile, the APP5A^−/−^ mice had a lower threshold than the APP mice in response to mechanical stimulation (Fig. 3A), indicating that *Clec5a* knockout attenuates the mechanical nociception in the AD mouse model. In the hot plate test, APP mice required a higher temperature to induce paw withdrawal than WT mice, suggesting an impairment in thermal nociception. However, *Clec5a* knockout did not significantly alter the thermal-induced paw withdrawal threshold in the WT mice or the APP mice (Fig. 3B). These findings suggested that the absence of *Clec5a* rescues the deficits of mechanical nociception but not thermal nociception in the AD mouse model.

### *Clec5a* knockout does not impact locomotor and anxiety behavior

**Fig. 4:**
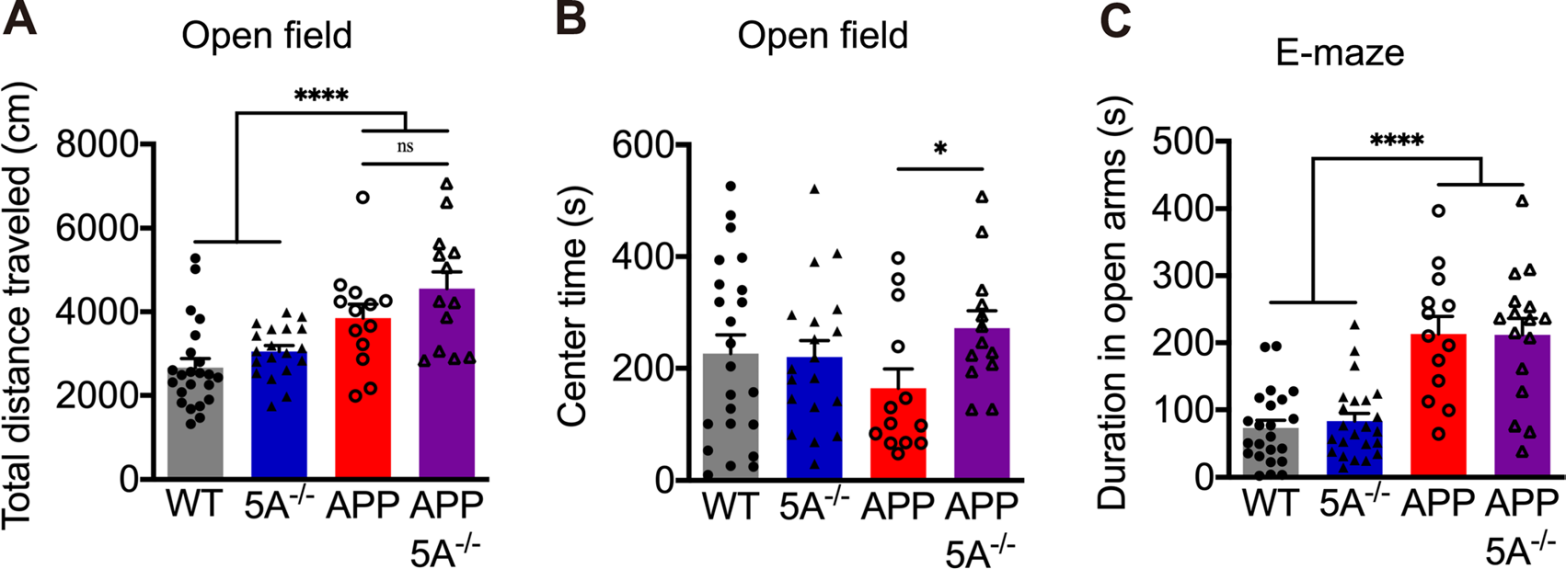
*Clec5a* knockout does not alter locomotor activity but partially ameliorates anxiety-related behaviors in the AD mouse model. (**A**,**B**) In the open field test, the traveled distance (**A**) evaluates locomotor activity, while the time spent in the center area of the chamber (**B**) measures anxiety levels. (**C**) In the elevated plus maze, the time spent in open arms measures the anxiety level. *n* = 23 for WT, *n* = 21 for 5A^−/−^, *n* = 13 for APP and APP5A^−/−^. *p ≤ 0.05, ****p ≤ 0.0001

In addition, we examined whether the *Clec5a* knockout affects locomotor and anxiety-like behaviors by the open field and elevated plus maze tests. This APP mouse model showed hyperactivity in the open field test and lower anxiety in the elevated plus maze [37]. In the open field test, APP mice traveled longer distances than WT mice, suggesting that APP mice have higher locomotor activities. However, the *Clec5a* knockout did not alter the locomotor activity in APP mice (Fig. 4A). The APP and WT mice did not show a significant difference in the time spent in the center area, but APP5A^−/−^ mice spent significantly more time than APP mice in the center area (Fig. 4B). In the elevated plus maze test, the APP mice spent significantly more time traveling in the open arms than WT mice (Fig. 4C), indicating that APP mice have lower anxiety-related behavior. However, APP5A^−/−^ mice did not significantly alter the open arm time compared with APP mice. In both tests, the performance of 5A^−/−^ mice was no different from WT mice. Therefore, deleting *Clec5a* did not alter locomotor activity, but its impact on anxiety-related behaviors requires further investigation.

### *Clec5a* knockout reduces Aβ deposition

**Fig. 5:**
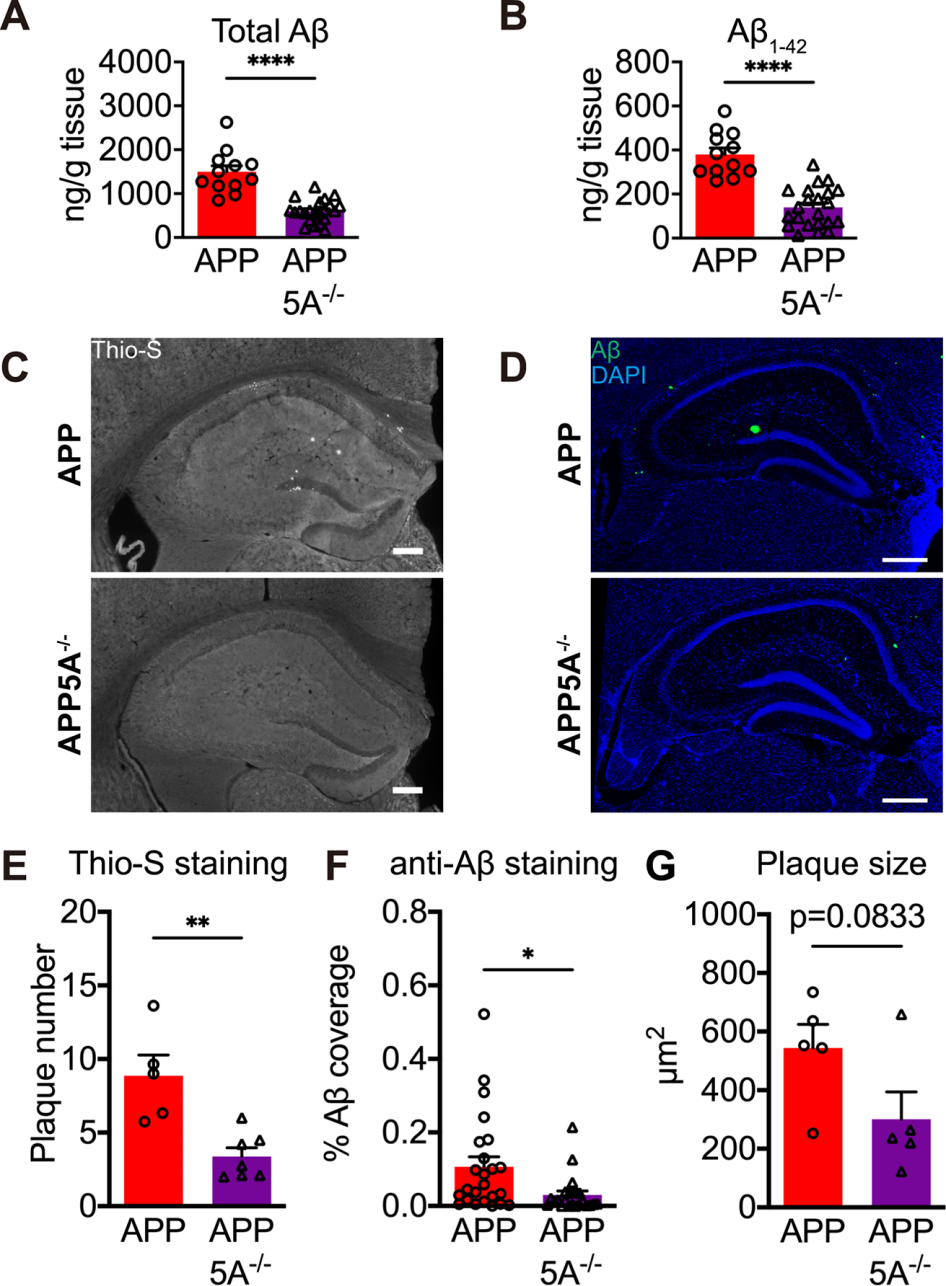
*Clec5a* knockout reduces the Aβ accumulation in the AD mouse model. (**A**, **B**) The levels of guanidine soluble total Aβ (**A**) and the Aβ_1−42_ (**B**) in mouse hippocampus were measured by ELISA. *n* = 12 for APP, *n* = 22 for APP5A^−/−^. (**C-F**) Amyloid plaques were analyzed using Thioflavin-S (Thio-S) and anti-Aβ antibody staining. Representative images of Thioflavin-S^+^ staining (**C**) and Aβ antibody staining (**D**). The number of Thioflavin-S^+^ plaques (**E**) and the percent area covered by plaques (**F**) in the hippocampus. 3–7 slices per mouse and 5–7 mice in each genotype were analyzed. Scale bar: 200 μm. (**G**) The size of each plaque was determined from the Aβ antibody staining. *n* = 88 plaques for APP, *n* = 110 plaques for APP5A^−/−^ from 5 mice in each genotype. *p ≤ 0.05, **p ≤ 0.01, ****p ≤ 0.0001

Aβ deposition is one of the pathological markers of AD. These mice were sacrificed after behavioral tests to examine whether *Clec5a* knockout alters Aβ levels. ELISA was used to examine the guanidine-soluble Aβ levels in the hippocampus of APP and APP5A^−/−^ mice. Both total Aβ and Aβ_1−42_ levels in the hippocampus were significantly lower in APP5A^−/−^ mice than in APP mice (Fig. 5A, B).

To investigate amyloid plaque distribution in the hippocampus, brain slices from APP and APP5A^−/−^ mice were stained with Thioflavin-S (Thio-S) to detect amyloid plaque with β-sheet conformation (Fig. 5C) and with anti-Aβ antibody to detect total Aβ deposition (Fig. 5D). In the Thio-S staining, the number of amyloid plaques was significantly decreased in the hippocampus (Fig. 5E). Furthermore, the area of total Aβ deposition was measured by anti-Aβ antibody staining (Fig. 5D). The area covered by Aβ in the hippocampus was significantly lower in APP5A^−/−^ mice than in APP mice (Fig. 5F). In addition, the plaque size showed a downtrend in APP5A^−/−^ mice (Fig. 5G). These results revealed that the *Clec5a* knockout reduces the Aβ deposition in the AD mouse model.

### *Clec5a* knockout increases plaque-associated microglia

**Fig. 6:**
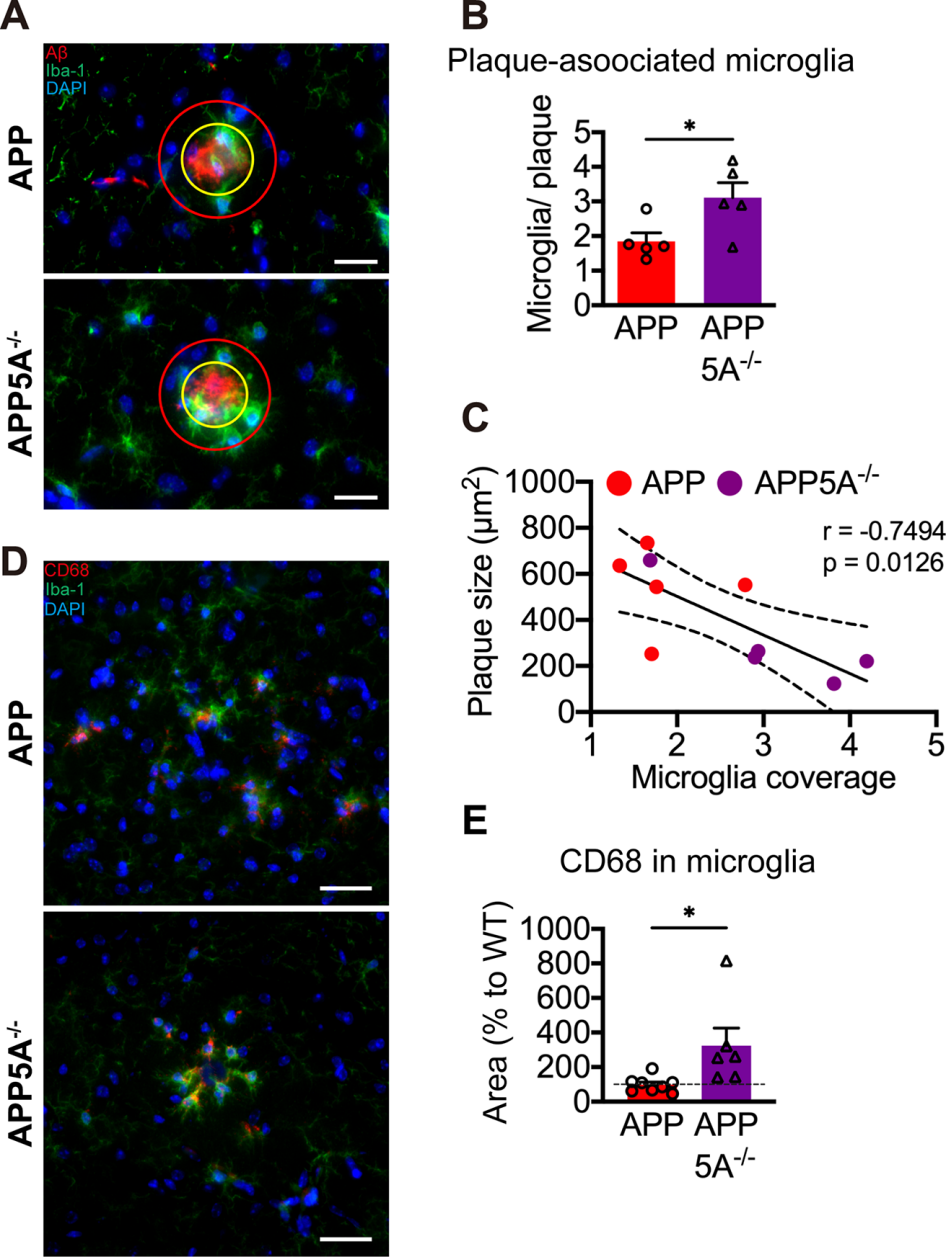
*Clec5a* knockout increases the plaque-associated microglia in the AD mouse model. (**A**) The methodology for assessing the coverage of plaque-associated microglia labeled with Iba1 (*green*) and Aβ (*red*) antibodies. DAPI (*blue*) was used to indicate the nuclei. The yellow circle represents the region covering the entire Aβ plaque. The red circle, expanded by a 10 μm radius from the yellow circle, represents the plaque-associated area. Scale bar: 20 μm. (**B**) The plaque-associated microglia coverage was calculated as the ratio of the microglia area in the yellow circle to the plaque area in the red circle. (**C**) The correlation between plaque-associated microglia coverage and plaque size was determined using Pearson correlation analysis. Dashed line: 95% confidence interval bands. Solid line: best-fit line. *n* = 88 plaques for APP, *n* = 110 plaques for APP5A^−/−^ from 5 mice in each genotype. (**D**) The representative images of CD68 *(red)* and Iba-1 *(green)* double staining. DAPI (*blue*) was used to indicate the nuclei. Scale bar: 30 μm. (**E**) The microglial CD68 area was calculated and normalized to the WT group. *n* = 8–14 slices for each mouse. 8 mice for the APP group and 6 mice for the APP5A^−/−^ group were analyzed. *p ≤ 0.05

Microglia are recruited to amyloid plaques in AD patients and mouse models. These plaque-associated microglia are hyperreactive for immune response and phagocytosis [42]. The plaque-associated microglia, which is defined as microglia within 10 μm from the edge of plaque, were analyzed in the hippocampus of APP and APP5A^−/−^ mice (Fig. 6A). The ratio of microglia coverage around the plaque was significantly increased in APP5A^−/−^ mice (Fig. 6B). We analyzed the correlation between the size of the plaque and the microglia coverage around the plaque. The plaque size was negatively correlated with the microglia coverage in the hippocampus (Fig. 6C). The increase in plaque-associated microglia is not due to the increase in the total number of microglia. We found that the density of microglia in the hippocampus had no significant difference among all four genotypes (Fig. S2A, B). Upon Aβ stimulation, microglia usually change their morphology by shortening their processes and enlarging their cell body [43]. The coverage of microglial cells in the hippocampus was significantly higher in APP mice than WT mice, but it was lower in APP5A^−/−^ mice than in APP mice (Fig. S2C). Moreover, the average size of microglia is significantly smaller in the APP5A^−/−^ mice than in APP mice (Fig. S2D), suggesting the reduction of activating microglia in *Clec5a* knockout.

Plaque-associated microglia have direct access to amyloid plaque, which they remove through phagocytosis. Thus, we examined the level of CD68, a marker of phagocytosis, in APP and APP5A^−/−^ mice. The level of CD68 in the microglia was significantly higher in APP5A^−/−^ mice than in APP mice (Fig. 6D, E). These results indicated that the *Clec5a* knockout in APP mice increased the recruitment of microglia to amyloid plaques and implied the increasing phagocytosis of Aβ in microglia.

### *Clec5a* knockout alters the inflammatory signals

**Fig. 7:**
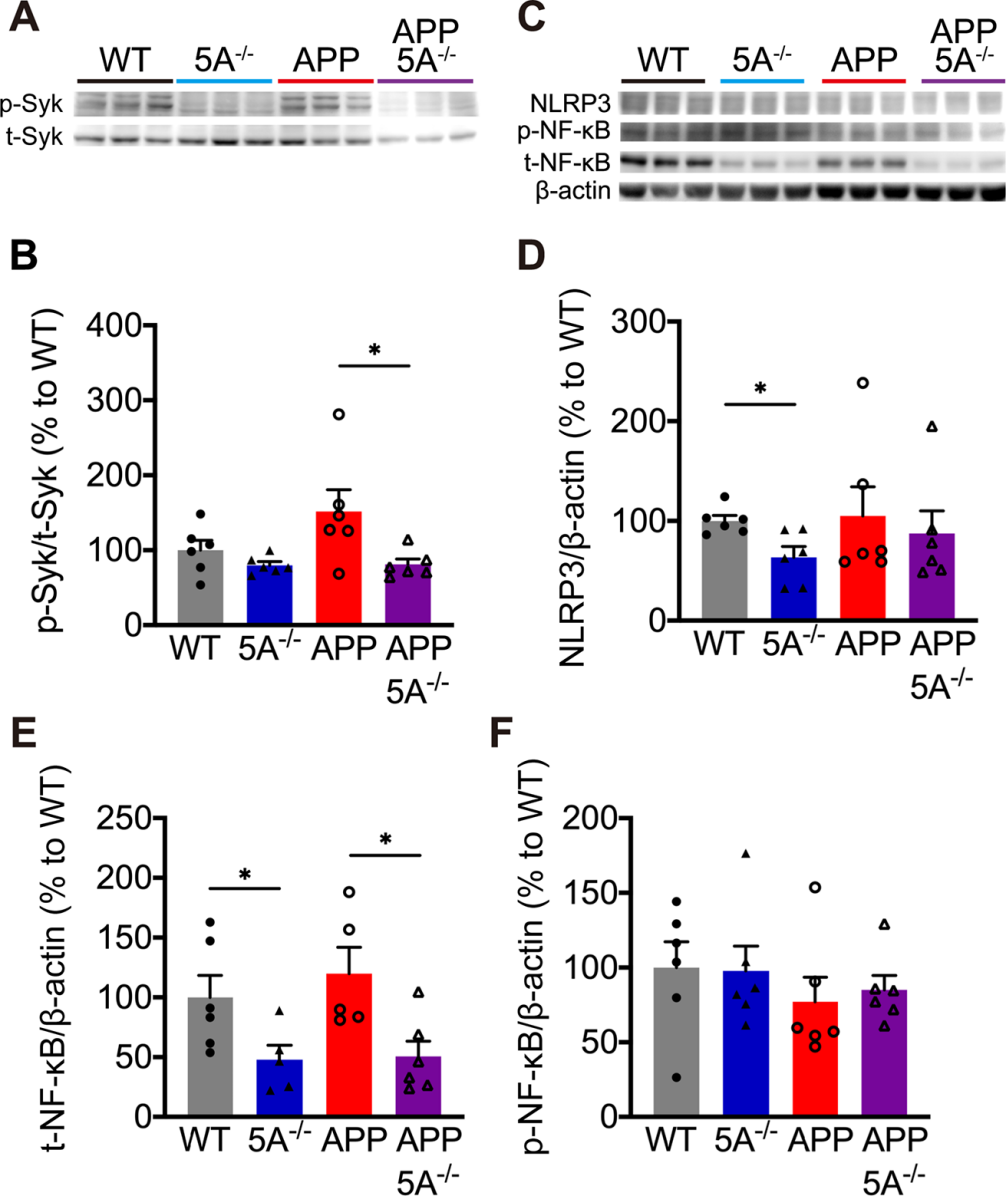
The inflammatory signals were altered in the hippocampus of *Clec5a* knockout mice. (**A**, **B**) The Syk activation was measured by western blotting and calculated as the ratio of phosphorylated Syk (p-Syk) to total Syk (t-Syk). (**C-F**) The level of NLRP3 (**D**), total NF-κB (t-NF-κB) (**E**), and phosphorylated NF-κB (p-NF-κB) (**F**) were normalized to β-actin. *n* = 5–6 mice for each genotype. *p ≤ 0.05

Multiple inflammatory response pathways might be modulated by CLEC5A. CLEC5A can activate the Syk signaling, which has been implicated in the regulation of Aβ production and neuroinflammation [44, 45]. Using Western blot analysis, the ratio of phosphorylated Syk is significantly lower in APP5A^−/−^ mice than in APP mice (Fig. 7A, B). Previous studies indicated that CLEC5A regulates the NLRP3 inflammasome activation [35, 46]. Our Western blot analysis indicated that the level of NLRP3 is lower in 5A^−/−^ mice than in WT mice (Fig. 7C). The expression of NLRP3 is regulated by NF-κB, which could be activated by Syk signaling in macrophages [47, 48]. In the hippocampus, knockout *Clec5a* significantly reduced the total protein level of NF-κB (Fig. 7E), but no difference in phosphorylated NF-κB level (Fig. 7F). However, we did not detect significant alteration in other potential targets, such as AKT (Fig. S3A), and the cytokines, such as IL-1β, IL-18, IL-6, and IL-10 (Fig. S3B-E) in these mice.

### *Clec5a* knockdown enhances phagocytosis and inhibits pro-inflammatory response in vitro

**Fig. 8:**
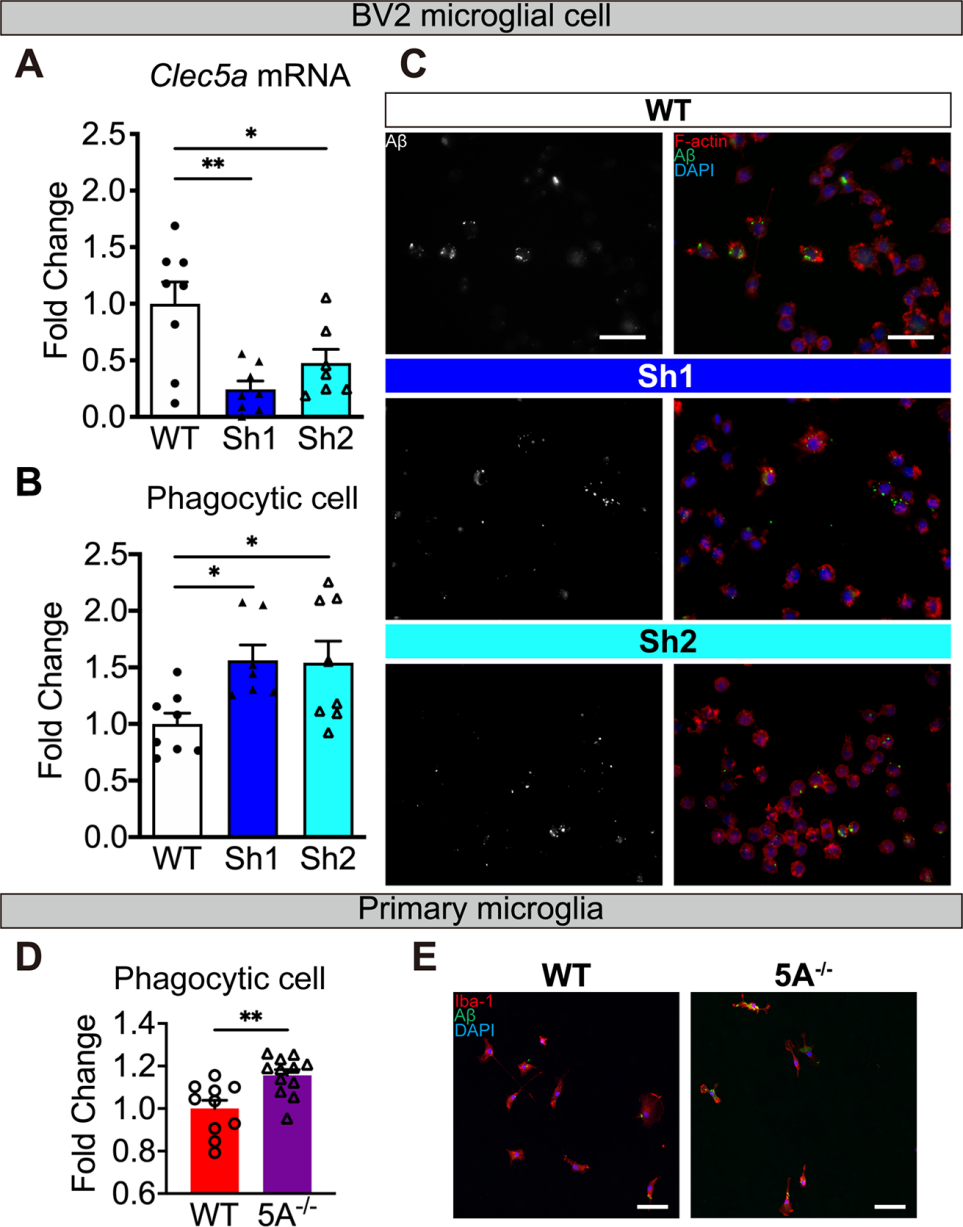
Reduction of *Clec5a* increases Aβ phagocytosis in BV2 microglial cells and primary microglia. (**A**) The *Clec5a* levels in 2 different *Clec5a* shRNA expression stable cell lines (Sh1 and Sh2) were determined by qPCR. *n* = 8 wells for each group. (**B-E**) The BV2 cells (**B**, **C**) and primary microglia (**D**, **E**) were stimulated with oAβ for 24 h and then incubated with fluorescent conjugated Aβ for another 24 h. The ratio of phagocytic cells was calculated as the number of Aβ containing cells divided by the number of total cells. For BV2 lines, *n* = 7–8 views per line from at least 3 independent experiments. For primary microglia, *n* = 10 wells for WT and *n* = 12 wells for 5A^−/−^. (**C**) The representative phagocytosis images of F-actin (*red*), fluorescent conjugated Aβ (*green*), and cell nuclei (*blue*). (**E**) The representative phagocytosis images of Iba-1 (*red*), fluorescent conjugated Aβ (*green*), and cell nuclei (*blue*). Scale bar: 50 μm. All data were normalized to WT cells. *p ≤ 0.05, **p ≤ 0.01

To knockdown *Clec5a in vitro*, we generated two stable BV2 microglial lines with two different shRNAs (Sh1 and Sh2, Fig. 8A). The reduction of Aβ deposition in APP5A^−/−^ mice may be due to the enhancement of microglial phagocytosis. To monitor the phagocytosis efficiency, the oligomeric Aβ-stimulated BV2 cells were incubated with the fluorescence-conjugated Aβ for 24 h. The percentage of Aβ-colocalized cells was higher in both *Clec5a* knockdown cells (Fig. 8B, C). These results suggested that Aβ-induced phagocytosis is increased upon *Clec5a* knockdown. Similarly, the primary microglia generated from 5A^−/−^ mice also had higher Aβ phagocytosis ability than from WT mice (Fig. 8D, E). This finding aligns with the decrease of Aβ deposition observed in vivo (Fig. 5C-F).

The blockade of CLEC5A has been reported to decrease *Nlrp3* expression after virus infection [35]. To compare with the in vivo finding, we investigated the expression of *Nlrp3* upon *Clec5a* knockdown after 24 h of Aβ treatment. In WT cells, the level of *Nlrp3* mRNA was higher in Aβ treated cells than in untreated cells. In both *Clec5a* knockdown cell lines, the levels of Aβ-induced *Nlrp3* mRNA were significantly reduced (Fig. S4A). Next, we examined if *Clec5a* modulates the Aβ-induced pro-inflammatory response in microglia. The level of pro-inflammatory cytokines TNF-⍺ in the Aβ conditioned media and *Il-6* mRNA level was lower in *Clec5a* knockdown cells than in the WT cells (Fig. S4B, C).

## Discussion

This study demonstrated that CLEC5A mediates the functional and pathological deficits in AD development. In the APP transgenic mouse model, the *Clec5a* knockout ameliorates memory and nociception deficits. In their hippocampus, the *Clec5a* knockout decreases the amyloid deposition, increases plaque-associated microglia, and raises CD68 signal in microglia, suggesting the increase of phagocytosis in APP5A^−/−^ mice. Furthermore, the reduction of *Clec5a* increases Aβ phagocytosis in vitro. Therefore, CLEC5A deficiency enhances microglial recruitment and phagocytosis, resulting in decreased plaque burden and ameliorated cognitive deficits.

*Clec5a* knockout attenuated Aβ burden in our AD mouse model, potentially due to increasing plaque-associated microglia and promoting microglial phagocytosis. The activated microglia are recruited to the plaque site to eliminate Aβ aggregates and to create a barrier to impede the spread of Aβ [49–51]. The increase of microglia coverage surrounding the plaque reduces plaque size and dystrophic neurite [52]. These microglia could engulf the Aβ into lysosome and thus reduce amyloid plaques [53–55]. In agreeing with this possibility, APP5A^−/−^ mice had an increase of CD68 signal in the microglia. CD68 is a lysosomal glycoprotein shuttling between lysosomes, endosomes, and the plasma membrane [56]. The expression of CD68 is low in resting microglia and high in phagocytic microglia [57, 58]. Thus, *Clec5a* knockout increases phagocytotic microglia in APP mice. In parallel, both *Clec5a* knockout primary cell and *Clec5a* knockdown in the microglia cell line had better Aβ phagocytosis ability. In combination, our results indicated that *Clec5a* knockout enhances phagocytosis to remove Aβ.

In this study, the NLRP3 protein level is reduced in the *Clec5a* knockout mouse, and the *Nlrp3* mRNA level is reduced in the *Clec5a* knockdown microglia culture. Similarly, the virus-triggered CLEC5A activates the NLRP3 inflammasome to mediate the inflammatory signals [35, 59]. Upon virus infection, CLEC5A triggers the phosphorylation of Syk to propagate pro-inflammatory responses [25, 30, 60]. In this study, APP mice with *Clec5a* knockout had a lower ratio of Syk phosphorylation. Syk is an activator of the NF-κB signaling pathway to control the inflammatory response in macrophages [61]. In AD pathogenesis, Aβ can activate Syk signaling to induce the transcription and the assembly of the NLRP3 inflammasome [27]. Inhibition of NLRP3 can alleviate AD-like functional deficits and pathological features both in vitro and in vivo [62]. The NLRP3-dependent inflammatory response requires two steps-priming and activation [63]. The priming step induces the production of the NLRP3 protein mediated by NF-κB. The activation step includes the inflammasome assembly and autoproteolytic activation of downstream substrates. Our in vivo result found the reduction of the NF-κB and NLRP3 protein levels but no significant change of IL-1β and other cytokines. Thus, the impact of *Clec5a* knockout may alter at the priming stage.

*Clec5a* deficiency attenuates the behavioral symptoms in AD pathology. Besides memory deficits, approximately 45% of AD patients experience abnormal pain perception, frequently exhibiting reduced sensitivity to pain [64–66]. Our previous research showed that APP mice have reduced mechanical and thermal nociceptive sensitivities due to the aberrant activation of striatal-enriched protein tyrosine phosphatase (STEP) signaling in the hippocampus [38]. In the present study, the deficiency of *Clec5a* restored the responsiveness to mechanical pain but not thermal pain. Similarly, suppressing microglial pro-inflammatory activity attenuates pain tolerance and hyperalgesia in the neuropathic pain rodent model [67–69]. Furthermore, the central mechanisms governing thermal and mechanical nociception differ in several inflammatory-related nociceptive responses [70–73]. Our finding suggests that alongside the STEP signaling pathway, modulating microglial activation may also contribute to the altered mechanical pain perception observed in the AD mouse model.

## Conclusion

In summary, lowering CLEC5A levels positively impacts the disease progression in AD models. While the mechanism requires further investigation, reducing the microglial CLEC5A could enhance the Aβ clearance in the AD model. Furthermore, this study implies that blocking CLEC5A with the monoclonal antibody [31,75] may serve as a therapeutic strategy for AD.

## Materials and methods

### Animal

The *APP* transgenic mouse line J20 (B6.Cg-Zbtb20^Tg(PDGFB−APPSwInd)20Lms^/2Mmjax) was used in this study as an AD mouse model. This transgenic mouse line expresses a human *APP* minigene with the Swedish (K670N/M671L) and Indiana (V717F) familial AD mutations. The hemizygous h*APP* transgenic mice express high levels of Aβ in the mice brain [74]. The *Clec5a* knockout mouse, in which exons 3 to 5 of *Clec5a* were excised, was generated in a previous study [30]. The genotype of these mice was verified by PCR.

### Microglia isolation

The 6-month-old and 10-month-old mice were sacrificed to isolate their hippocampi. The hippocampal tissues were homogenized on the Single cell suspension dissociator (DSC-400; RWD) by enzymatic digestion with cell debris removal kit (DHABE-5003; RWD). The microglia were purified by incubating with CD11b MicroBeads (130-093-634; Miltenyi Biotec) and separated by MACS separator (MiniMACS™ Separator; Miltenyi Biotec).

### Morris water maze

The Morris water maze was performed to assess the learning and memory in mice. The mice were placed in a rounded pool (122 cm in diameter) with a platform (14 cm in diameter) submerged 1 cm below the water surface. The 5-day hidden platform test consisted of 10 sessions (2 per day). In each session, the mice were allowed to explore the pool in three 60-second trials with 15-minute inter-trial intervals. The escape latency serves as the index for memory acquisition. For each day, the entry points were changed semi-randomly. The probe trial was conducted one day after the last session of hidden platform training by removing the platform and allowing the mice to explore the maze for 1 min. The time spent in the target quadrant, where the platform had previously been located, was used to measure memory retention. The time spent in the target quadrant and the swim speed were recorded and analyzed with the EthoVision video tracking system (Version 3.1, Noldus).

### Elevated plus maze

The elevated plus maze consisted of an elevated plus-shape apparatus with two open arms and two closed arms. The mice were placed individually at the center of the maze and allowed to explore the maze freely for 10 min. The time spent on each arm was recorded and calculated using the EthoVision video tracking system.

### Open field test

The open field test was performed using an automated Flex-Field/Open field Photo-beam Activity System (Version 2.0, TRU Scan Photobeam LINC, Coulbourn Instruments), which is a clear plastic open chamber (41 × 41 × 38 cm) with 16 × 16 infrared photo-beam arrays placed 1.5 cm above the bottom of the chamber. Mice were placed in this chamber for 15 min, and the arena’s beam breaks were counted.

### von Frey filaments test

Mice were placed on an elevated rack with meshes for 30 min for habituation. Several von Frey filaments (VFF) with different forces (0.07, 0.16, 0.4, 0.6, 1.4, 4, and 6 g; NC12775; North Coast Medical) were applied to the hind paws of these mice using the up-and-down method. The withdrawal responses were determined by the lift of their stimulated paw.

### Hot plate test

Mice were placed on a thermal apparatus with an analgesia meter (Model PE34; IITC Life Science). The surface of the plate was heated from 30℃ with an increase of 6℃ per minute until the temperature reached 55℃. The temperature was recorded when the withdrawal responses were observed. The withdrawal responses were determined by the lift or lick of their hind paws.

### Enzyme-linked immunosorbent assay (ELISA)

For measuring Aβ levels, the mice hippocampal tissues were homogenized in 5 M guanidine/5 mM Tris buffer (pH 8.0). The samples were diluted in 0.25% casein-blocking buffer containing 0.5 M guanidine and protease inhibitor (04693116001; Roche) before applying to total Aβ and Aβ_1−42_ ELISA kits (27729, 27711; IBL). For measuring the murine IL-1β, the mice hippocampal tissues were homogenized in RIPA buffer (RB4477; Bio Basic) containing protease and phosphatase inhibitors (04693132001, 04906837001; Roche). The homogenates were applied to the IL-1β ELISA kit (432604; BioLegend). The concentration was determined from the standard curve and normalized to tissue weight according to the manufacturer’s protocol.

### Multi-Plex immunoassay

Frozen mice cortical tissues were homogenized with lysis buffer (150 mM NaCl, 7.6 mM NaH_2_PO_4_, 32.4 mM Na_2_HPO_4_, 1% Triton-X) containing protease and phosphatase inhibitors. The levels of murine IL-6, IL-10, and IL-18 of cortical lysates were measured by Multi-Plex immunoassay service in the Inflammation Core Facility, Academia Sinica, Taiwan.

### Western blotting

The mice hippocampal tissues were isolated and homogenized with RIPA buffer (RB4477; Bio Basic) containing protease and phosphatase inhibitors. The total protein concentration in tissue homogenates was quantified by BCA assay (23225; Thermo Scientific), and 30 µg of total protein was used in the western blot. The protein samples were mixed with SDS sample buffer and 2-Mercaptoethanol (M6250; Sigma-Aldrich) and boiled to 98℃ for 15 min. The boiled protein samples were then separated via 10% Tris-glycine SDS-polyacrylamide gel electrophoresis and transferred to PVDF membranes (IPVH00010; Merck Millipore). The membranes were probed with rabbit anti-Syk (#13198; Cell Signaling Technology), rabbit anti-p-Syk (AF8404; Affinity Biosciences), mouse anti-NLRP3 (AG-20B-0014-C100; AdipoGen Life Sciences), rabbit anti-NF-κB (#8242; Cell Signaling Technology), rabbit anti-p-NF-κB (#3033; Cell Signaling Technology), rabbit anti-AKT (#9272; Cell Signaling Technology), rabbit anti-p-AKT (#9271; Cell Signaling Technology), and mouse anti-actin (66009-1-Ig; Proteintech Group) antibodies. The membranes were washed and probed with the HRP-conjugated goat anti-mouse IgG and goat anti-rabbit IgG antibodies (115-035-003, 111-035-003; Jackson ImmunoResearch). Protein signals were developed using a chemiluminescent substrate ECL detection system (CCH345; BIO-HELIX) and quantified by using a luminescence imaging system (LAS-4000; Fujifilm).

### Immunohistochemistry (IHC) and Thioflavin-S staining

The brain was fixed by 4% paraformaldehyde (PFA) for 24 h and dehydrated in gradient sucrose buffer (10%, 20%, and 30% in sterile water; 7, 4, and 12 h for each concentration, respectively). The brain is embedded in optimal cutting temperature compounds (Tissue-Tek^®^ O.C.T. Compound; Sakura) and frozen by liquid nitrogen. The tissues were sectioned coronally in 30 μm slices using a sliding microtome (CM1900; Leica). The brain slices were stored in cryoprotectant medium (30% glycerol, 30% ethylene glycol in PBS) at -20 °C. For IHC, the brain slices were blocked by phosphate-buffered saline (PBS) containing 10% FBS (fetal bovine serum, 35-010-CV; Corning) and 0.5% Triton X-100 (TB0198; Bio Basic). The slices were stained with primary antibodies including anti-Aβ antibody (D54D2, #8243; Cell Signaling Technology or 6E10; 803014; BioLegend), anti-Iba-1 antibody (019-19741; Wako), anti-CLEC5A antibody (FAB1639R; R&D Systems), and anti-CD68 antibody (ab31630; Abcam) for 24 h in 4℃. The CF^®^ Dye labeled secondary antibodies conjugated with different fluorescents (20303, 20019, 20023; Biotium) were applied to sections for 1 h at room temperature. For the Thioflavin-S staining, the slices were incubated with 0.015% Thioflavin-S (T1892; Sigma-Aldrich) for 20 min at room temperature. After mounting, the brain slices were imaged using a Zeiss fluorescence microscope (Axio Observer A1; Zeiss).

### Image analysis

The number of Thioflavin-S^+^ plaque and microglia density in the hippocampal area were counted manually. The coverage of Aβ was defined as the percentage of Aβ area in the hippocampal area. The microglia coverage was defined as the percentage of Iba-1 positive area normalized to the hippocampal area. The microglial CD68 expression was defined as the percentage of CD68 positive area in the hippocampal Iba-1 positive area. The Aβ, Iba-1, CD68, and hippocampal area were measured by ImageJ software (ImageJ; NIH). To analyze the plaque-associated microglia, a circular region encompassing the entire plaque was established. Subsequently, the circle was expanded by a radius of 10 μm. Microglia within this enlarged circle were identified as plaque-associated, and their area was quantified using ImageJ software (ImageJ; NIH).

### Cell culture

Mouse microglial BV2 cell line was maintained in RPMI 1640 medium (50-020-PB; Corning) supplemented with 10% fetal bovine serum (FBS; 35-010-CV; Corning) and incubated at 37℃ in 5% CO_2_.

### Single cell-derived *Clec5a* knockdown BV2 cell lines

The plasmids containing *Clec5a* shRNAs (Table 1) were purchased from the National RNAi Core Facility, Academia Sinica, Taipei, Taiwan. The plasmid containing *Clec5a* shRNA was transfected into BV2 cells by Lipofectamine 2000 (11668500; Invitrogen) for 4 h and then recovered in RPMI 1640 medium (50-020-PB; Corning) supplemented with 10% fetal bovine serum (FBS; 35-010-CV; Corning) for 24 h. To select transfected cells, 5 µg/ml puromycin (P8833; Sigma-Aldrich) was applied to the medium for 24 h. Single colonies were picked and diluted in a 96-well plate with RPMI + FBS. The levels of *Clec5a* in each clone were measured by qPCR. Two stable *Clec5a*-knockdown cell lines (Sh1, Sh2) were selected for in vitro study.

**Table 1:** Sequences of *Clec5a* shRNA

Sh1	CCGGCGCTGGATCTGCGAAATGAATCTCGAGATTCATTTCGCAGATCCAGCGTTTTTG
Sh2	CCGGCTGAAGTATCTTCAGGACATACTCGAGTATGTCCTGAAGATACTTCAGTTTTTG

### Primary microglia culture

Cortices from the postnatal day 0–5 mice were dissected in ice-cold Hank’s Balanced Salt Solution media (HBSS; 20-021-CVR; Corning). Tissues were dissociated by Single Cell Suspension Dissociator (DSC-410; RWD Life Science) with High Activity Neonatal Brain Enzymatic Digestion Kit (DHNBE-5002; RWD Life Science), and then the dissociated cells were collected by a cell strainer (130-110-916; Miltenyi Biotec). The cells were centrifuged at 2400×g at room temperature and re-suspended by Dulbecco’s Modified Eagle Medium/Nutrient Mixture F-12 media (DMEM/F12; 12400024; Gibco) containing 10% FBS and 1% Penicillin-Streptomycin solution (CC502-0100; GeneDireX). The cells were seeded on the 25 cm² Cell Culture Flask (PC272-0200; GeneDireX) and renewed the culture medium every 3 days. After the cells reached the confluency, the cells were treated with 0.25% trypsin solution (CC511-0100; GeneDireX) in DMEM/F12 medium without serum at 37℃ for 10 min to obtain the microglia-enriched cells. Next, the microglia-enriched cells were detached with 1.25% trypsin solution (CC511-0100; GeneDireX) in PBS at 37℃ for 1 h. The microglia cells were centrifuged at 1800 ×g for 10 min and seeded at the number of 2 × 10^4^ cells on poly-D-lysine (P7280; Sigma-Aldrich) pre-coated coverslip.

### Oligomeric Aβ (oAβ) preparation

For preparing the oAβ, HFIP-treated Aβ_1−42_ peptides (A-1163-2; rPeptide Inc.) were dissolved in 10% Dimethyl sulfoxide (DMSO, 97063-136; VWR International) at 100 µM and stored at -80℃. The oAβ was generated by incubating the Aβ stock at 4℃ for 24 h, snap frozen by liquid nitrogen, and storing at -80℃.

### Phagocytosis assay

For measuring the phagocytosis, the primary microglia cells and BV2 cells were activated by 5 µM oAβ for 24 h and then followed by incubation with 0.2 µM fluorescent-conjugated Aβ_1− 42_ peptide (AS-60479-01; AnaSpec). After 24 h, cells were fixed by 4% PFA. The primary microglia cells were stained with anti-Iba-1 antibody (019-19741; Wako) followed by the CF^®^ Dye labeled secondary antibody (20019; Biotium). The BV2 cells were stained with fluorescent-conjugated phalloidin (#00045; Biotium). The cells were imaged by the Zeiss fluorescence microscope (Axio Observer A1; Zeiss), and the percentage of phagocytic microglial cells was analyzed by ImageJ software (ImageJ; NIH).

### Secreted TNF-⍺ measurement

The WT and *Clec5a*-knockdown BV2 microglial cells were treated with 5 µM oAβ for 24 h. The cultured media were diluted and applied to the TNF-⍺ ELISA kit (430916; BioLegend) according to manufactural instructions. TNF-⍺ concentration was determined from the standard curve and normalized to the total cell number.

### Quantitative PCR (qPCR)

The RNA was extracted from the isolated microglia and BV2 cells using GENEzol™ Reagent (GZR100; Geneaid). The purified RNA was reverse-transcribed into cDNA using the High-Capacity RNA-to-cDNA™ Kit (4387406; Applied Biosystems). The cDNA was mixed with qPCRBIO SyGreen Blue Mix Hi-ROX (PB20.16; PCR Biosystems Inc.) and analyzed by a StepOnePlus Real-Time PCR System (Applied Biosystems). *Iba-1* was used as the internal control for measuring the *Clec5a* level in adult microglia, whereas the *Gapdh* was used as the internal control for measurements of *Clec5a*, *IL-6*, and *Nlrp3* in BV2 cells. The sequences of primers are listed in Table 2. The data was analyzed using StepOne software version 2.0.

**Table 2:** Sequence of primers for qPCR

Name	Sequence
*Clec5a*	Forward: CCGAGCAGGAGCATACATTCA
	Reverse: GGGGACGAAGCCATCATTAC
*Gapdh*	Forward: GCATCCACTGGTGCTGCC
	Reverse: TCATCATACTTGGCAGGTTTC
*Iba-1*	Forward: GGATCAACAAGCAATTCCTCG
	Reverse: AACTCCATGTACTTCACCTTGA
*Il-6*	Forward: ACCACGGCCTTCCCTACTTC
	Reverse: TCTGTTGGGAGTGGTATCCTCTGT
*Nlrp3*	Forward: GGCCTTCAGGCTGATCCAA
	Reverse: TAGCCCCGTGCACACAATC

### Statistical analysis

Data are presented as the mean ± s.e.m. Statistical analyses were performed with GraphPad Prism (Version 8.0; GraphPad). Differences among multiple means were assessed by one-way or two-way ANOVA, followed by Tukey’s post-hoc test. Unpaired t-tests analyzed differences between two means and the inter-group comparison of behavior tests. Pearson correlation analysis was used to analyze the correlation between Aβ plaques and microglia coverage, and linear regression was applied to determine the confidence interval of the best-fit line. The threshold for significance was defined as *p* < 0.05.

## Electronic supplementary material

Below is the link to the electronic supplementary material.

Supplementary Material 1

Supplementary Material 2

## Data Availability

No datasets were generated or analysed during the current study.

## Abbreviations

Aβ: β-Amyloid
AD: Alzheimer’s disease
APP: Amyloid precursor protein
CLEC5A: C-type lectin domain family 5 member A
ELISA: Enzyme-linked immunosorbent assay
GAPDH: Glyceraldehyde-3-phosphate dehydrogenase
Iba-1: Ionized calcium-binding adaptor molecule 1
IL-1β: Interleukin-1β
IL-6: Interleukin-6
NF-κB: Nuclear factor kappa-light-chain-enhancer of activated B cells
NLRP3: NOD-like receptor protein 3
qPCR: Quantitative PCR
STEP: Striatal-enriched protein tyrosine phosphatase
Syk: Spleen tyrosine kinase
Thio-S: Thioflavin-S
TNF-⍺: Tumor necrosis factor ⍺
