# Supplementary material for: The deficient CLEC5A ameliorates the behavioral and pathological deficits via the microglial Aβ clearance in Alzheimer’s disease mouse model
Source: J Neuroinflammation. 2024 Oct 23;21:273. doi: 10.1186/s12974-024-03253-x (PMC11515658; doi:10.1186/s12974-024-03253-x)
Supplement: Supplementary file 1 — Supplementary Material 1 [file 12974_2024_3253_MOESM1_ESM.pdf]

Supplementary Figures

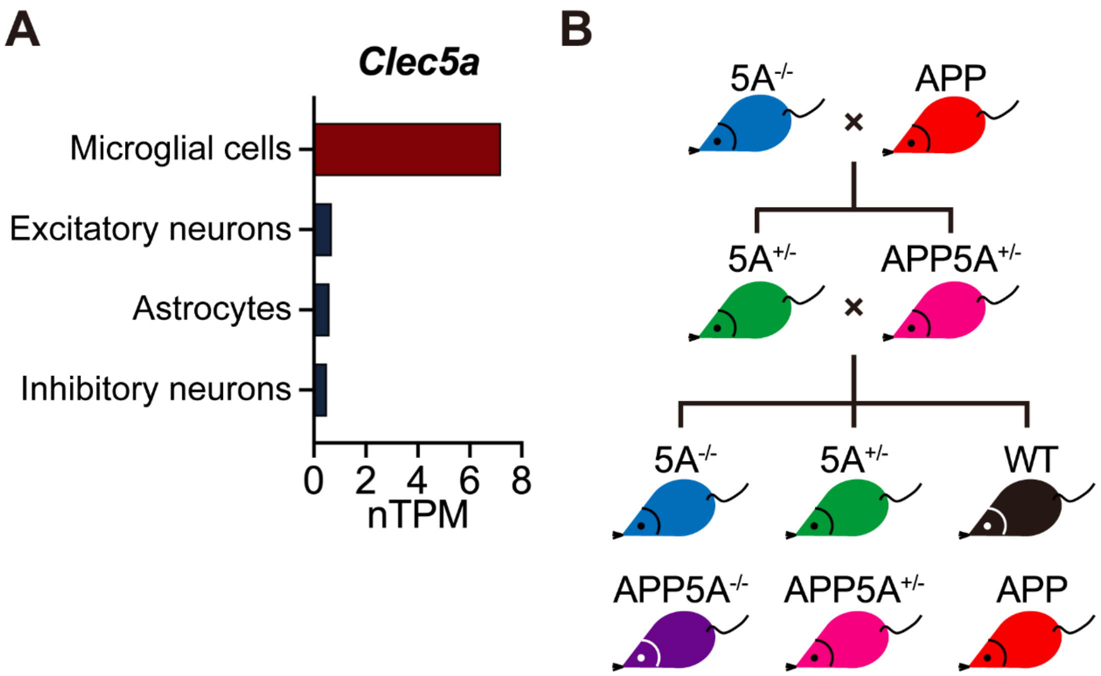

Figure S1. (A) The *Clec5a* is mainly expressed in microglial cells in the mouse brain based on the online database, The Human Protein Atlas, Protein Atlas version 23.0 (proteinatlas.org). **(B)** Breeding schematic to generate APP mice with *Clec5a* knockout and other 5 genotypes of littermate controls.

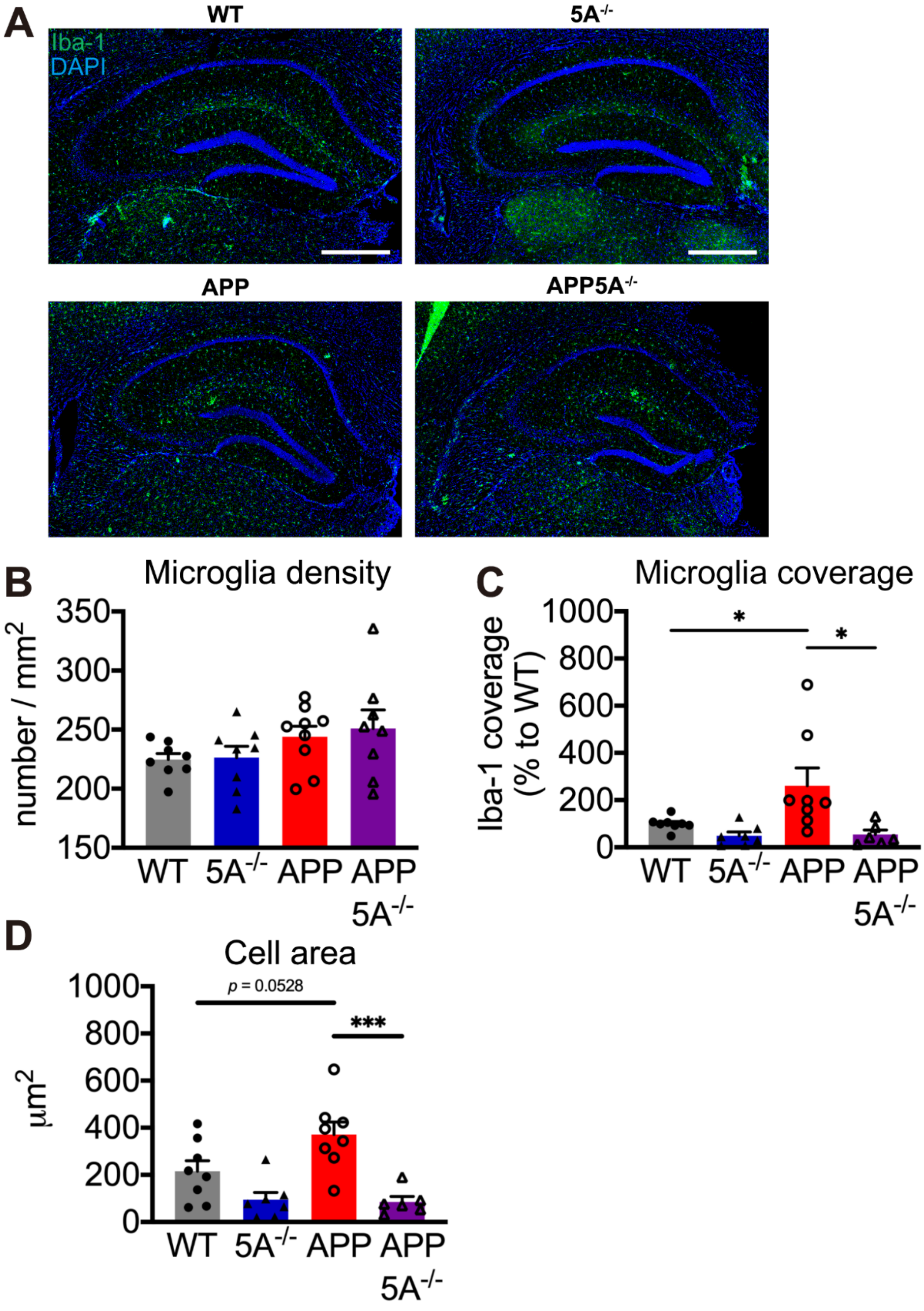

Figure S2. The *Clec5a* knockout altered the microglial coverage and cell size, but not cell density, in the hippocampus. **(A)** The representative images of hippocampal sections staining with Iba-1 staining *(green)* and DAPI *(blue)*. Scale bar: 500 μm. **(B)** The microglia density in the hippocampus was calculated as number/mm^2. **(C)** The microglia coverage was calculated as the Iba-1$^+$ area normalized to the hippocampus area. The result in the WT group was set as 100. **(D)** The average area of each microglia in the hippocampus. n = 8-14 slices per mouse and 7-9 mice for each genotype. *$p \leq 0.05$, ***$p \leq 0.001$.

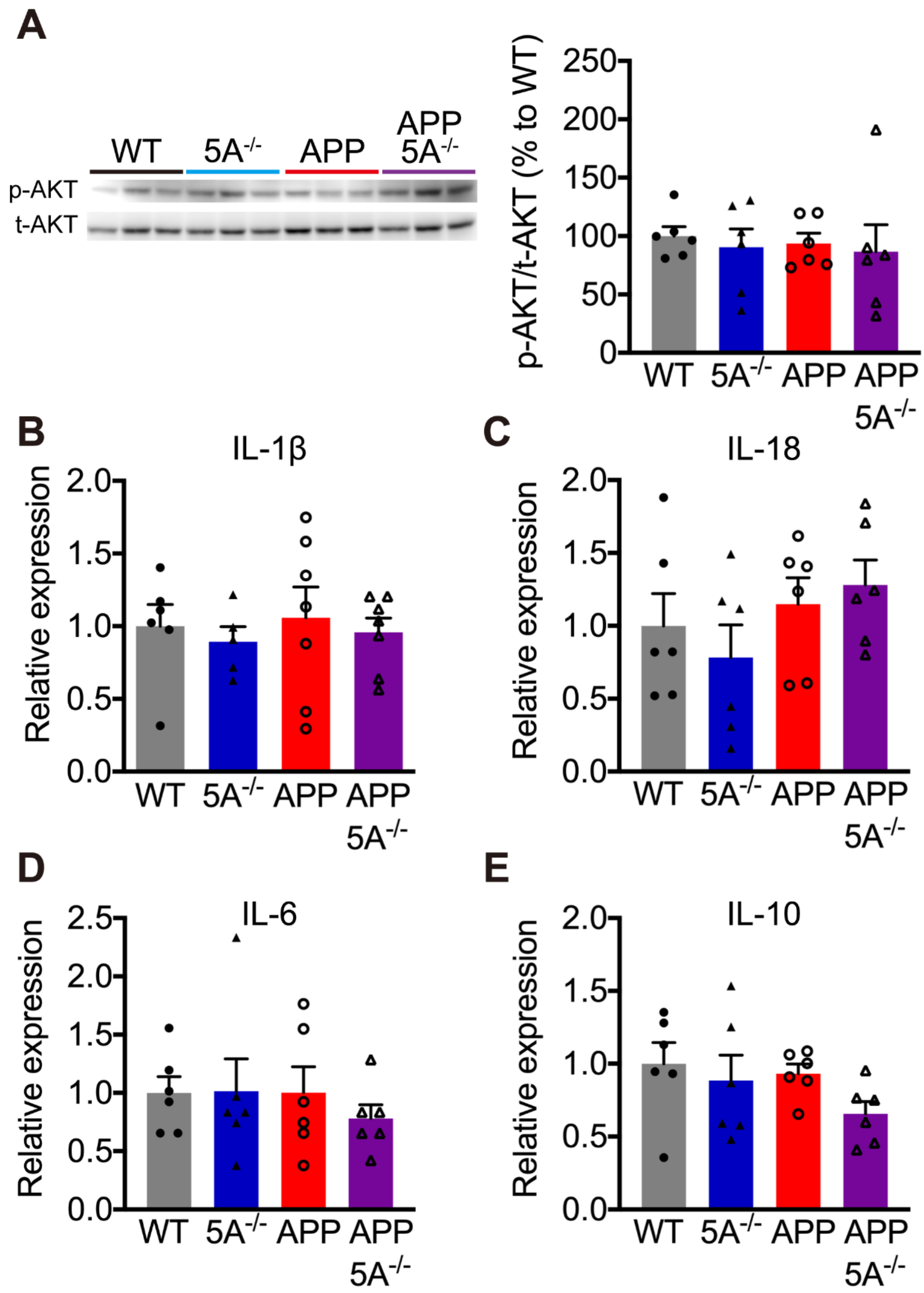

Figure S3. The AKT activation and inflammatory cytokines were not altered in *Clec5a* knockout mice. **(A)** The AKT phosphorylation was measured by western blotting. The activity of AKT was calculated by the ratio of p-AKT to t-AKT. n = 6 mice for each genotype. **(B)** The IL-1β protein level in mice hippocampus was measured by ELISA and normalized to the WT group. n = 5-7 mice for each genotype. **(C-E)** The protein levels of IL-18 **(C)**, IL-6 **(D)**, and IL-10 **(E)** were measured by the Multi-Plex immunoassay. n = 6 mice for each genotype.

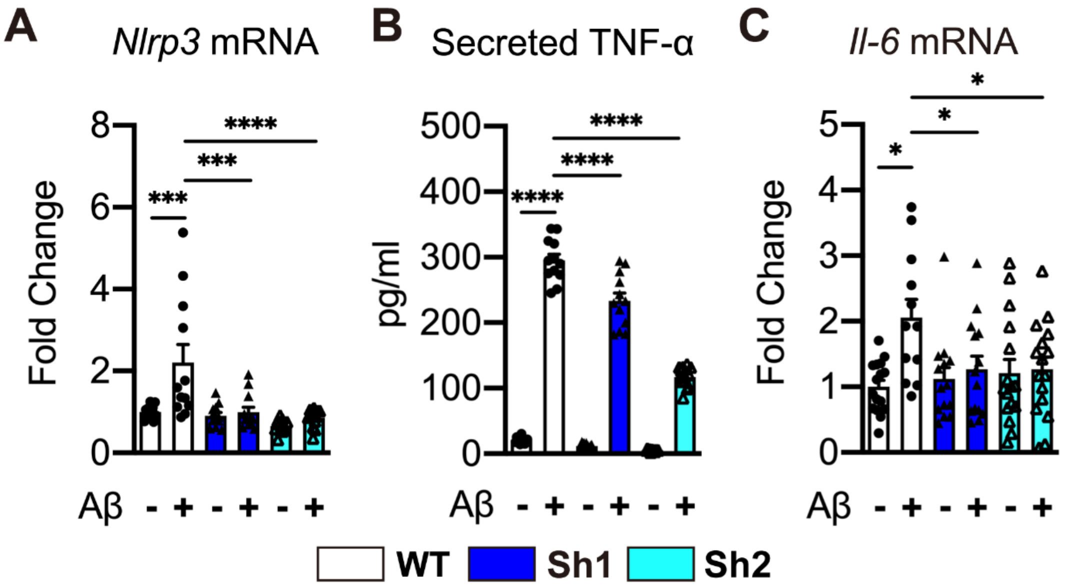

Figure S4. *Clec5a* knockdown decreases the Aβ-induced inflammatory signals in BV2 microglial cells. The BV2 cells (WT, Sh1, Sh2) were treated with or without oAβ for 24 hours. **(A)** The mRNA level of *Nlrp3* in the treated cells was analyzed by qPCR. n = 12-16 wells from 4 independent experiments in each group. **(B)** The level of TNF-α secreted from the cells was examined by ELISA. n = 12 wells from 3 independent experiments in each group. **(C)** The mRNA level of *Il-6* in the treated cells was analyzed by qPCR. n = 12-16 wells from 4 independent experiments in each group. All data were normalized to the WT without oAβ treatment group. **p* ≤ 0.05, ****p* ≤ 0.001, *****p* ≤ 0.0001.
