# Supplementary material for: The deficient CLEC5A ameliorates the behavioral and pathological deficits via the microglial Aβ clearance in Alzheimer’s disease mouse model
Source: J Neuroinflammation. 2024 Oct 23;21:273. doi: 10.1186/s12974-024-03253-x (PMC11515658; doi:10.1186/s12974-024-03253-x)

Full images of western blotting

Blots were imaged and quantified using a luminescence imaging system (LAS-4000; Fujifilm). To minimize the interruption between different targets, the membranes for p-AKT and t-AKT blots (Fig S3) were cropped at the molecular weight (MW) around 50 kDa. The membranes for NLRP3, NF-κB, and β-actin blots (Fig 7) were cropped at the MW around 75 kDa and 50 kDa. The membranes were stripped (15 g Glycine, 1 g SDS, 10 ml Tween-20 in 1 L deionized water) and blocked (10% milk in TRIS-buffered saline) between phosphorylated protein and total protein blots. The red boxes indicate the representative cropped images used in Figures 7 and Figure S3.

Figure 7

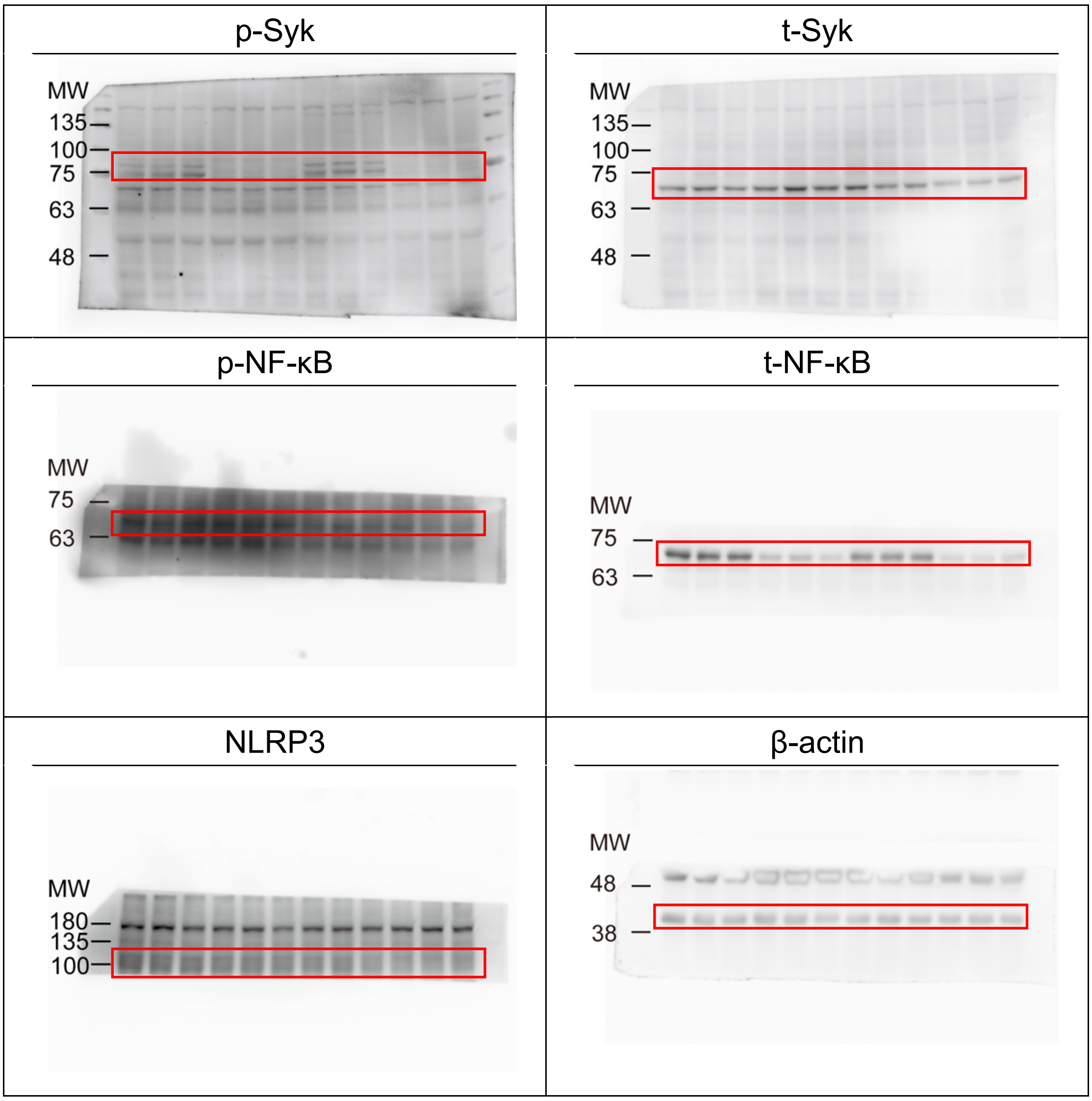

p-Syk
MW
135
100
75
63
48
t-Syk
MW
135
100
75
63
48
p-NF-κB
MW
75
63
t-NF-κB
MW
75
63
NLRP3
MW
180
135
100
β-actin
MW
48
38

Figure S3

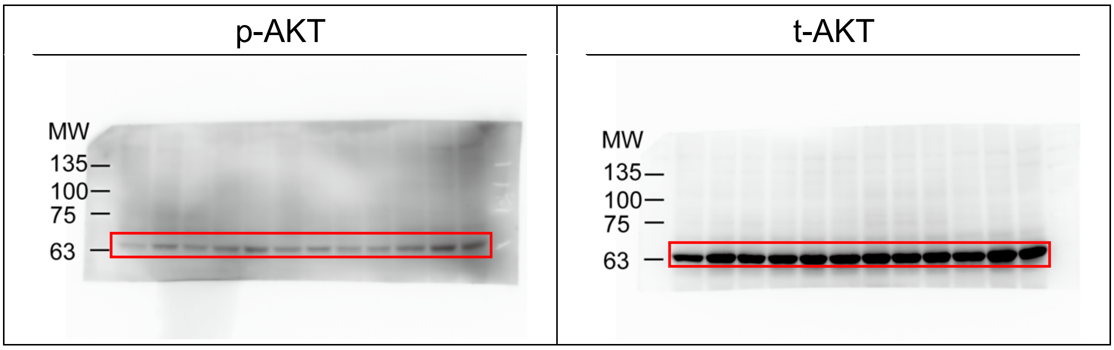

Supplement: Supplementary file 2 — Supplementary Material 2 [file 12974_2024_3253_MOESM2_ESM.pdf]
